# Automated Analysis of Acetaminophen Toxicity on 3D HepaRG Cell Culture in Microbioreactor

**DOI:** 10.3390/bioengineering9050196

**Published:** 2022-05-02

**Authors:** Martin Baca, Dana Brauer, Maren Klett, Uta Fernekorn, Sukhdeep Singh, Jörg Hampl, G. Alexander Groß, Patrick Mai, Karin Friedel, Andreas Schober

**Affiliations:** Department of Nano-Biosystems Engineering, Institute of Chemistry and Biotechnology, University of Technology, P.O. Box 10 05 65, 98684 Ilmenau, Germany; baca.martin2@gmail.com (M.B.); maren.klett@tu-ilmenau.de (M.K.); uta.fernekorn@web.de (U.F.); sukhdeep.singh@tu-ilmenau.de (S.S.); joerg.hampl@tu-ilmenau.de (J.H.); alexander.gross@tu-ilmenau.de (G.A.G.); maipatrick@outlook.de (P.M.); friedel.gehren@freenet.de (K.F.)

**Keywords:** flow-through ELISA, 3D hepatocyte culture, HepaRG, albumin, acetaminophen toxicity, scaffold

## Abstract

Real-time monitoring of bioanalytes in organotypic cell cultivation devices is a major research challenge in establishing stand-alone diagnostic systems. Presently, no general technical facility is available that offers a plug-in system for bioanalytics in diversely available organotypic culture models. Therefore, each analytical device has to be tuned according to the microfluidic and interface environment of the 3D in vitro system. Herein, we report the design and function of a 3D automated culture and analysis device (3D-ACAD) which actively perfuses a custom-made 3D microbioreactor, samples the culture medium and simultaneously performs capillary-based flow ELISA. A microstructured MatriGrid^®^ has been explored as a 3D scaffold for culturing HepaRG cells, with albumin investigated as a bioanalytical marker using flow ELISA. We investigated the effect of acetaminophen (APAP) on the albumin secretion of HepaRG cells over 96 h and compared this with the albumin secretion of 2D monolayer HepaRG cultures. Automated on-line monitoring of albumin secretion in the 3D in vitro mode revealed that the application of hepatotoxic drug-like APAP results in decreased albumin secretion. Furthermore, a higher sensitivity of the HepaRG cell culture in the automated 3D-ACAD system to APAP was observed compared to HepaRG cells cultivated as a monolayer. The results support the use of the 3D-ACAD model as a stand-alone device, working in real time and capable of analyzing the condition of the cell culture by measuring a functional analyte. Information obtained from our system is compared with conventional cell culture and plate ELISA, the results of which are presented herein.

## 1. Introduction

Three-dimensional organotypic cell culture devices are gradually taking over the standard 2D cell culture as an experimental model for mammalian biology and disease. In particular, 3D models such as spheroids, body-on-a-chip (including stem cell research) and dynamic in vitro models are increasingly accepted by organizations such as the OECD (Organization for Economic Co-operation and Development) [1,2]. Various disciplines of science and technology are joining this venture to create such systems with the highest common features of real organs, including engineered 3D extracellular environments and vascularization and, most importantly, automation. With advancement in micromachining, engineering and microfluidics, it is possible to design systems with minimal human involvement which, in turn, offer reduced risks of contamination and human error. Currently, organotypic 3D models range up to millifluidic scale systems [3,4], where most systems are based on static, passive fluidics [5,6,7]. However, dynamic (perfused) fluidic systems offer significant advantages, for example, increased transport of nutrients and metabolites within the 3D culture [8,9,10]. The fluid flow also has a direct influence on cell proliferation [11]. In principle, external pumping devices can be connected to most of the modern chamber-based cultivation systems. However, precise programing and chip-based components can provide much better control over not only pumping but also reversing the flow direction that can be utilized for sampling, which is an important factor when considering stand-alone systems without human interfaces.

Perfused cell culture systems, in particular, for the liver, are important for long-term experiments, where cell health or functional markers can be monitored without disassembling the culture system. Since the structures of most body-on-a-chip systems are very complex, online monitoring is desirable for this purpose. Therefore, a number of next-generation liver-on-a-chip systems have been developed that are equipped with biosensors or bioimaging, enabling the online monitoring of pH and oxygen [12,13], cellular metabolic state [14,15] and the detection of cell-derived analytes in the culture medium [16,17,18]. This way, cells do not need to be removed from the perfused culture systems to identify drug toxicity and cellular health. In particular, quantitative analysis of cell-secreted proteins by microfluidic ELISA provides a suitable method for measuring non-invasively the drug toxicity in complex culture systems [16]. However, most studies use PDMS (polydimethylsiloxane)-based chip technology which strongly interferes with the detection of soluble marker proteins due to adsorption [19].

Despite the growing trend for developing integrated microfluidic cell culture systems (among companies such as InSphero AG, Schlieren-Zürich, CH and MIMETAS, Leiden, The Netherlands), this field is still in its infancy due to the huge technical differences in modern cultivation systems developed in each lab. For example, hydrogel-based 3D in vitro systems suffer from poor mechanical stability and lengthy chemical modification, while spheroids, which have been mainly used for 3D cell culture and extensively characterized [20,21], are not suitable for fluidic applications. Currently, there is no plug-in analytical and fluidic device that can be universally adapted. Therefore, each research laboratory working in this area is developing an automated culturing and analysis device that suits the purpose. Over the last decade, we have developed a modern cultivation device with a designated 3D cell culture MatriGrid^®^ scaffold [22]. A family of microbioreactors has been engineered to meet the microfluidic and biosensoric needs specific to our research purpose to design disease models for the liver. However, an automated fluidic setup for culturing and analysis for the system is desirable.

Therefore, we developed a PDMS-free, fully automated device (the “3D Automated Culture and Analysis Device”, 3D-ACAD for short) that combines a 3D hepatocyte cell culture with automated perfusion, medium change, repeated drug application and sampling. Using this system, it is possible to perform a microfluidic albumin ELISA (“on-demand ELISA”) to assess hepatotoxicity without disassembling or opening the entire culture and analysis system. We preferred to develop a scaffold-based 3D culture system that allows good access of the drug to the cells with minimal adsorption and absorption of small molecules, drugs and biomolecules. The perfusion experiments were performed with HepaRG cells, as this cell line expresses many drug-metabolizing genes (CYP proteins, transporters, nuclear receptors) at a level similar to that of primary human hepatocytes [23]. The present paper first provides a short characterization (albumin production, cell health, polarity and CYP expression) of the scaffold-based 3D differentiated HepaRG culture and compares it with a differentiated monolayer culture (2D). Subsequently, we show the results of an albumin ELISA performed with the 3D-ACAD system in which the HepaRG cultures were perfused with acetaminophen (APAP)-supplemented medium over a period of 96 h. We demonstrate that 3D cultures of HepaRG cells are comparable to 2D monolayer cultures in terms of hepatocyte function (albumin, MRP-2 drug transport activity and CYP expression) and polarity (ZO-1), making them suitable for toxicity studies. Furthermore, we show that 3D-ACAD can be used to evaluate the hepatotoxicity of drugs using albumin measurements. Our study shows that this highly integrated in vitro system can be used for drug toxicity testing (at least for preclinical risk assessment) and that it provides a useful alternative model for assessing hepatotoxicity by incorporating 3D HepaRG cultures.

## 2. Materials and Methods

### 2.1. 3D Cell Carrier MatriGrid^®^

**Figure 1:**
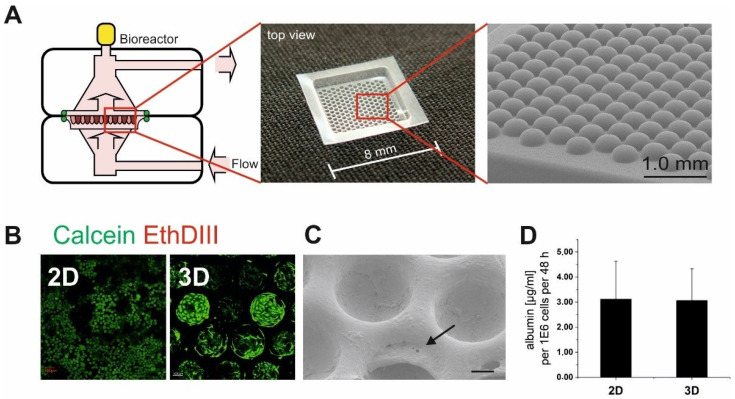
(**A**) schematic illustration of the bioreactor of the culture unit with integrated MatriGrid^®^ scaffold for 3D culturing, with medium flow direction marked. (**B**) Live/dead staining of 2D (monolayer)- and 3D (MatriGrid^®^)-cultured HepaRG cells after 28 days differentiation. Living cells are visualized by calcein dye (green), dead cells by ethidium DIII (red). The scale bar corresponds to 100 µm. (**C**) SEM image of differentiated 3D (MatriGrid^®^)-cultured HepaRG cells. The scale bar corresponds to 100 µm. The arrow indicates a membrane-like structure at the top of the cavity. (**D**) Average albumin production (hepatocyte functional marker) of monolayer (2D)- and 3D-cultured HepaRG cells after 28 days differentiation. Mean values ± SE are shown; n = 3.

Three-dimensional organotypic cell culturing was performed in a porous polycarbonate scaffold named a MatriGrid^®^ (Figure 1A). The scaffold contains 187 microcavities to culture the cells. Fabrication and quality control of the scaffolds have been previously described in detail [22]. In summary, the scaffold consists of a rectangular 50-micron thick biocompatible polycarbonate (PC) piece with a microstructured seeding area of 5 × 5 mm^2^. Porous polycarbonate foils are structured with our microthermoforming technique by means of which an appropriate pore size limited to the cell culturing cavity is achieved [24]. MatriGrids^®^ generated by this method are necessary for the nutrient supply in the active perfusion of 3D-cultured cells inside the bioreactor.

### 2.2. 3D-ACAD—Culture Unit

The culture unit (shown in the results section; for detailed information see Supplementary Materials, Culture Unit) accommodates the bioreactor with the MatriGrid^®^ and the cell culture, which is actively perfused using an integrated pump. In addition, it changes the culture medium and extracts medium samples at preprogrammed intervals. The culture unit is intended to operate in a CO_2_ incubator at 37 °C. It was constructed with the following properties: utilization of the existing in-house microbioreactor and MatriGrid^®^ scaffolds (Figure 1A), active perfusion of the 3D cell culture and automated medium change and sampling of the culture medium for the purpose of analysis, with optional sample dilution. The fluidic network of the culture unit is divided into two parts (Figure S1, Supplementary Materials). The first part comprises the circulation loop, the bioreactor and the fresh medium reservoir. It is required to work under aseptic conditions; therefore, this part is removable. The cell culture, supported on the MatriGrid^®^, can be inserted into the bioreactor and the whole circulation loop can be filled with culture medium under the clean bench. The second part of the fluidic network was designed to handle the sample or the waste medium from the bioreactor and sterile operating conditions are not required. To avoid biomarker absorption or adsorption on the surface of fluidic pathways, the design avoids using strongly absorbing materials such as PDMS. The optimal flowrate for the perfusion of 3D-grown HepaRG culture on the MatriGrid^®^ was found to be 12 µL/min.

### 2.3. Chemicals and Reagents

Acetaminophen (APAP), collagen, penicillin/streptomycin, Williams’ Medium E, hydrocortisone hemisuccinate, insulin, dimethyl sulfoxide (DMSO), DAPI (4′,6-Diamidino-2-phenylindole dihydrochloride), Mowiol and 6-carboxyfluorescein diacetate were purchased from Sigma-Aldrich (Steinheim, Germany); fetal bovine serum and glutamine were purchased from Biochrom (Berlin, Germany). For immunofluorescence staining, the following antibodies were used: anti-ZO1 mouse IgG (BD Biosciences, Heidelberg, Germany), anti CYP2E1 rabbit and anti CYP3A4 rabbit IgG (Bioss Antibodies, Woburn, MA, USA), AlexaFluor594 labeled goat anti-mouse and AlexaFluor488 labeled goat anti-rabbit antibody (Invitrogen, Darmstadt, Germany). A live/dead cell staining kit II was purchased from Promocell.

### 2.4. Cell Culture

HepaRG cells were provided by BIOPREDIC International, Saint Grégoire (France). Undifferentiated cells were grown for maintenance in Williams’ Medium E containing 10% fetal bovine serum (FBS), 5 µg/mL insulin, 5 × 10^−5^ M hydrocortisone hemisuccinate, 2 mM glutamine, 100 U/mL penicillin and 100 µg/mL streptomycin at 37 °C in a cell incubator at 95% relative humidity and 5% CO_2_.

### 2.5. HepaRG 2D and 3D Culture

For individual experiments, HepaRG cells were seeded at a density of 50,000 cells per well either in collagen pre-coated (5 µg/cm^2^) 24-well plates (monolayer) or collagen-pre-coated MatriGrid^®^ scaffolds (3D organotypic cell culture) in 24-well plates. The desired cells for the monolayer culture were seeded in 1 mL of culture medium per well. To ensure selective growth in the microcavities of the MatriGrid^®^, cells were seeded in a small volume of medium (25 µL) and were allowed to adhere for 2 h before adding the remaining culture medium (1 mL) into the wells. After seeding, cells were cultured for 2 weeks in the maintenance medium. Thereafter, cells were shifted to differentiation medium on day 14 (supplemented with 1% DMSO). Medium was renewed every 2 days.

### 2.6. Cell Number and Viability

The cell number and viability of HepaRG cells was determined using an Innovatis CASY^®^ Cell Counter + Analyzer system. After trypsination, 50 µL of cell suspension was mixed with 10 mL of Casyton solution and the total cell number (live and dead cells) and viability was determined, measuring cell membrane integrity by the impedance method. The cell viability after 96 h of HepaRG cells cultured in 2D or perfused in 3D-ACAD was determined by averaging the viability of cells that were adhered and cells that were to be found in the medium supernatant.

### 2.7. 6-Carboxyfluorescein Diacetate Excretion (MRP-2 Transporter Activity)

After 28 days of differentiation, HepaRG cells grown on coverslips (2D) or in scaffolds (3D) were incubated with 1 µg/mL 6-carboxyfluorescein diacetate (6-CFDA) in Williams’ medium E for 30 min at 37 °C in an incubator. Subsequently, cells were washed three times with phosphate-buffered saline (pH 7.4) and efflux of 6-CFDA into bile canalicular structures was captured using an OLYMPUS laser scanning microscope FV1000 (Olympus, Hamburg, Germany).

### 2.8. Live/Dead Staining

After 28 days of differentiation, HepaRG cells grown on coverslips (2D) or in scaffolds (3D) were incubated with WME medium contain 2 µM Calcein and 4 µM EthDIII for 45 min. Medium was discarded and fresh PBS solution was added. Images were captured with an OLYMPUS laser scanning microscope FV1000 (Olympus, Hamburg, Germany).

### 2.9. Immunofluorescence Staining

After 28 days of differentiation, HepaRG cells grown on coverslips (2D) or in scaffolds (3D) were washed twice with phosphate-buffered saline (PBS) and subsequently fixed for 15 min with 4% paraformaldehyde. After permeabilization with 0.25% Triton in PBS for 5 min, cells were blocked with 5% BSA in PBS for 30 min. Cells were incubated with the following antibodies overnight: mouse anti-human ZO-1, rabbit anti-human CYP2E1 and rabbit anti-human CYP3A4, followed by incubation with species-dependent secondary antibodies: Alexa Fluor 594-labeled goat anti-mouse antibody and Alexa Fluor 488-labeled goat anti-rabbit antibody. Cells on coverslips were mounted in Mowiol containing DAPI. Images were captured with an OLYMPUS laser scanning microscope FV1000 (Olympus, Hamburg, Germany).

### 2.10. SEM Imaging

Scanning electron microscopy (SEM) was used for the investigation of HepaRG cell adhesion on MatriGrids^®^ after 28 days of differentiation. Cells were fixed using 2.5% glutaraldehyde at 4 °C for 1 h and washed twice with A.dest. Samples were dried, sputtered with a thin platinum layer and examined by scanning electron microscopy (SEM Hitachi S 4800-II, Hitachi High-Technologies Europe GmbH, Krefeld, Germany).

### 2.11. Albumin ELISA

With components of a commercially available human albumin ELISA quantitation kit (Bethyl laboratories, Montgomery, TX, USA), albumin was measured in both MTP and 3D-ACAD devices. For the MTP assay, the instructions of the albumin ELISA quantitation kit were followed. For the 3D-ACAD assay, the optimized flow-through ELISA protocol was used (see Supplementary Materials, ELISA Automated Assay—Procedure, and Table S2: Timing of the complete assay sequence). Albumin concentrations were determined by comparison with a concurrently generated calibration curve in the range of 6.25–200 ng/mL. Albumin levels were normalized to the total cell number (per million cells). The QuantaRed™ Enhanced Chemifluorescent HRP Substrate (Life Technologies GmbH, Darmstadt, Germany, cat. no. 15159) was used as the substrate for the fluorescence measurement/quantitation by the 3D-ACAD device. TMB substrate was used for the MTP ELISA (Biomol, Hamburg, Germany, cat. no. ICT 6275).

### 2.12. Concentration-Dependent Effect of APAP on Albumin Secretion

After 4 weeks of differentiation, HepaRG cells grown in monolayer and in MatriGrid^®^ were incubated for 24 h with increasing concentrations of APAP (0, 1, 5, 10, 15, 20, 40 mM) in Williams’ medium E + 1% dimethyl sulfoxide, 0.1% FBS and 5 µg/mL insulin, 5 × 10^−5^ M hydrocortisone hemisuccinate, 2 mM glutamine, 100 U/mL penicillin and 100 µg/mL streptomycin (vehicle) in wells. APAP toxicity was measured by the determination of albumin secretion. Albumin levels in the culture supernatants were analyzed using the Albumin ELISA Quantitation kit and normalized to the total cell number (per million cells). Albumin secretion levels by HepaRG cells treated with APAP were normalized against the control value, i.e., without APAP, which was set to 100%.

### 2.13. 3D-ACAD—Analysis Unit

The analytic unit (shown in the results section, for detailed information, see Supplementary Materials, Analysis Unit) automatically performs the complete sandwich ELISA procedure (see Supplementary Materials—ELISA-Automated Assay Procedure). It works in connection with the cell culture unit, where the medium sample is first prepared and later transferred to the analytical unit to measure the biomarker concentration of interest. It utilizes an optimized flow-through ELISA protocol and uses PVC capillaries as the solid phase. During a single run, the device simultaneously measures 7 standards or samples within 3 h and 13 min. The implemented protocol comprises five steps: Firstly, the capillaries’ surfaces are coated with the capture antibody (Biomol, Hamburg, Germany, cat.No A80-129A-11), followed by incubation and washing. In the second step, free binding sites on the capillaries’ surface are blocked with BSA, followed by incubation and washing. Third, samples and standards are pumped to the capillaries. After the incubation, another washing step is included. In the fourth step, the HRP-conjugated secondary antibody (Biomol, Hamburg, Germany, cat.No A80-129P-30) is pumped to the PVC capillaries, then incubated and non-bound antibody is removed by a washing step. In the last step, the QuantaRed HRP™ substrate is transferred to the capillaries. After incubation, resorufin fluorescence is measured with integrated detection of the analysis unit (shown in results section, see Supplementary Materials—Fluorimeter, Excitation Optics, Emission Optics). The sequencing of the whole assay is controlled by the analyzer control unit (see Supplementary Materials—Control Unit). The design was optimized for efficient washing to prevent cross-contamination of the fluidic paths by different reagents (see Supplementary Materials—Washing Procedure). The C-Flex tubing was chosen for the fluidic connection due to its flexibility, low protein binding and chemical inertness to withstand aggressive cleaning. The fluidic path switching was realized by means of two-way and three-way solenoid pinch valves offering zero dead volume and biocompatibility of all wetted parts. Custom-made FEP manifolds with optimized topologies were used to achieve a high washing efficiency. The fluidics design avoids using strongly adsorbing or absorbing materials, such as PDMS.

Before each analytic run, the reagent containers were filled with corresponding reagents (washing buffer, capture antibody, blocking buffer, secondary antibody and the ADHP substrate), the containers for concentration standards were filled with corresponding albumin standards (0 ng/mL–50 ng/mL) and 7 PVC capillaries were installed into the sample changer (Figure S2). After the medium sample from the culture unit was available, the automated analysis procedure was started. After the measurement was completed, the automated cleaning procedure (see Supplementary Materials—Cleaning Procedure) was initiated, which requires 3 h and 25 min. After that, the analytic unit can be prepared for the next run.

### 2.14. Albumin ELISA with 3D-ACAD

After 4 weeks of static 3D culture, differentiated HepaRG cells in scaffolds were inserted into the microbioreactor filled with Williams’ Medium E (WME) with 0.1% FBS, 1% dimethyl sulfoxide, 5 µg/mL insulin, 5 × 10^−5^ M hydrocortisone hemisuccinate, 2 mM glutamine, 100 U/mL penicillin and 100 µg/mL streptomycin and perfused with an integrated peristaltic pump using the flow rate of 12 µL/min for 24 h to determine the basal albumin secretion of the cells. Subsequently, the cells were further perfused under the same conditions for another 72 h with the same medium or medium supplemented with APAP at a final concentration of 5 mM. Automatic sampling of culture medium containing albumin was undertaken every 24 h. Albumin levels were analyzed with 3D-ACAD and normalized later to the total cell number (per million cells) at each time point. The total cell count at each timepoint was determined from the cell count after the end of perfusion (after 96 h) and the cell counts that were to be found in the supernatant after each medium change. The albumin level at 24 h was set to 100% and albumin concentrations on the following 72 h were normalized to the value at 24 h.

### 2.15. Effect of APAP on Albumin Secretion of HepaRG Monolayer Cultures

Monolayer HepaRG cultures were differentiated in the same manner as described above. Upon completion, differentiated HepaRG cells were incubated with Williams’ medium E (WME) containing 0.1% FBS, 1% dimethyl sulfoxide, 5 µg/mL insulin, 5 × 10^−5^ M hydrocortisone hemisuccinate, 2 mM glutamine, 100 U/mL penicillin and 100 µg/mL streptomycin, initially for 24 h to determine the basal albumin secretion of the cells. Subsequently, the cells were incubated with the same medium or the medium supplemented with APAP at a final concentration of 5 mM for a further 72 h. The medium or the medium containing APAP was changed daily by hand. The medium was frozen and later analyzed for albumin content using an albumin quantification kit. In a parallel approach, cells were treated in the same manner, and cell counts of adherent cells and cells in the supernatant were determined after each medium change. Albumin levels were normalized to total cell counts (per million cells). Normalization was performed as described in Section: *Albumin ELISA with 3D-ACAD.*

### 2.16. Statistical Analysis

Significances (*p*-values) were estimated by the Student’s *t*-test.

Detailed information about the culture unit, analysis unit, control unit, fluorimeter and the washing procedure of the analysis unit can be found in the Supplementary Materials.

## 3. Results

### 3.1. Characterization of the 3D-Scaffold HepaRG Culture

The analysis of hepatotoxicity requires the selection of a suitable hepatocyte cell culture model with sufficient CYP expression, hepatofunctionality and polarity. Since the HepaRG cell line has phenotypic properties similar to primary hepatocytes [25,26,27], this cell type was selected for in vitro culturing and perfusion experiments. Consistent with the current opinion that 3D culture is more relevant in toxicity studies, culturing of HepaRG cells in MatriGrid^®^ scaffolds was chosen. The HepaRG cell line differentiates into mature hepatocytes and biliary cells and additional treatment with dimethyl sulfoxide induces CYP expression in this cell line. After completion of their differentiation, 3D HepaRG cultures, grown in collagen-coated porous polycarbonate scaffolds (MatriGrid^®^; Figure 1A) [22] were compared with the respective 2D monolayer cultures grown in collagen-coated polystyrene wells in terms of cell health (live/dead staining), hepatocyte functional markers (albumin, MRP-2 drug transport activity and CYP expression) and polarity/existence of *bile canaliculi* (ZO-1). For some experiments, HepaRG cells were cultured in parallel without the addition of the differentiating agent dimethyl sulfoxide for the same growth period.

**Figure 2:**
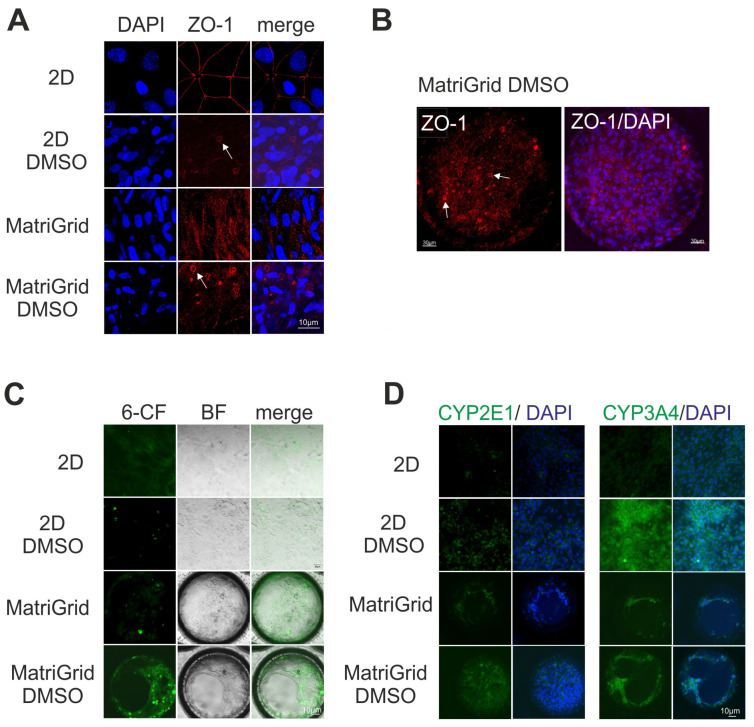
Immunofluorescence staining of markers of hepatofunctionality and polarity of monolayer (2D) and 3D HepaRG cells cultured without or with 1% dimethyl sulfoxide for 28 days. (**A**) The tight junctional marker ZO-1 labels bile pocket-like structures only in differentiated adjacent HepaRG-cells (visible as small holes between the hepatocytes and indicated by arrows). (**B**) Confocal image showing ZO-1 and DAPI staining in differentiated 3D (MatrGrid)-cultured HepaRG cells (arrows indicate *bile canaliculi*). (**C**) The 6-carboxy-fluorescein (6-CF) diacetate excretion for detection of drug transport and *bile canaliculi* functionality. (**D**) Detection of CYP2E1 and CYP3A4 in undifferentiated and differentiated HepaRG cells (drug metabolism). All the stainings were performed three times. BF: brightfield.

After the 4-week differentiation procedure, a good cell health of the HepaRG cells (monolayer and scaffold-cultured) was observed by live/dead staining (Figure 1B). Scaffold-cultured HepaRG cells showed a tissue-like growth consisting of stacks of cells; sometimes they formed membrane-like structures at the top of the cavities (Figure 1C). Due to the collagen-coating of the scaffold, this method of culturing HepaRG cells proved suitable for perfusion experiments, which require stable and adherent cell layers during the perfusion process and avoidance of cell detachment. Both 2D and 3D HepaRG cultures produced a sufficient amount of albumin, indicating good hepatocyte functionality after differentiation (Figure 1D). Reorganization of tight junctions was observed in differentiated HepaRG cells by immunofluorescence staining of *zona occludens* protein (ZO-1). Bile pocket-like structures were formed in differentiated 2D and MatriGrid^®^-cultured HepaRG cells (Figure 2A,B), which is a typical feature of this cell type [28,29]. In contrast, undifferentiated HepaRG cells (Figure 2A) were characterized by ZO-1 staining of adjacent membranes (2D-cultured HepaRG cells) or by a more cytoplasmatic localization of ZO-1 (MatriGrid^®^-cultured HepaRG cells). Moreover, accumulation of 6-carboxyfluorescein diacetate in these polarized structures confirmed the presence of *bile canaliculi* lumens between adjacent differentiated 2D- and MatriGrid^®^-cultured HepaRG cells and a functioning MRP-2 drug transport activity (Figure 2C).

To confirm whether the culture format affects the expression of acetaminophen (APAP)-metabolizing CYPs in HepaRG cells [30,31,32], the presence of CYP3A4 and CYP2E1 in cells without and with differentiation with DMSO was analyzed using immunofluorescence staining (Figure 2D). Both CYPs were clearly present after differentiation with DMSO in both 3D and 2D monolayer cultures. In contrast, 2D HepaRG cells cultured for 28 days without dimethyl sulfoxide seemed to have very weak expression of both CYP enzymes. Overall, these results suggest that the hepatotypic functionality of HepaRG cells, including cellular polarization, CYP expression and albumin release, is achieved in both culture formats after differentiation with dimethyl sulfoxide. We selected for our experimental setup the scaffold-based 3D culture with polarized cells, exhibiting both functional *bile canaliculi* and the presence of CYP2E1 and CYP3A4.

### 3.2. Automated Drug Treatment and Measurement of Biomarker Albumin

**Figure 3:**
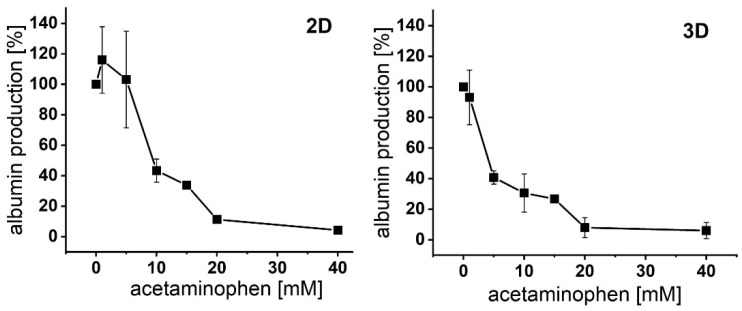
Concentration-dependent effect of acetaminophen on albumin production in HepaRG cells: monolayer- and MatriGrid^®^-cultured HepaRG cells were incubated with increasing concentrations of APAP (0, 1, 5, 10, 15, 20, 40 mM) for 24 h. Medium was analyzed for albumin concentration with a commercial ELISA quantitation kit. Albumin levels were normalized to the total cell number (per million cells). The albumin content of cells treated only with medium (control; 0 mM APAP) was set to 100% and the albumin production of cells treated with APAP was normalized to this control. The mean values ± SE are shown; n = 3 (2D monolayer), n = 4 (3D MatriGrid^®^) experiments.

A stable secretion of the analyte (Figure 1D) as well as its concentration-dependent reduction by liver-damaging drugs is a prerequisite for the medium analysis with the 3D automated culture and analysis device (3D-ACAD). Therefore, we investigated whether the potentially liver-toxic analgesic acetaminophen interferes with albumin synthesis in HepaRG cells in a concentration-dependent manner. Differentiated HepaRG cells grown as monolayer or cultured in MatriGrids^®^ were treated with increasing concentrations of acetaminophen for 24 h. Albumin concentrations in medium supernatants in response to APAP were analyzed by conventional MTP ELISA. We detected an impairment of albumin production in both monolayer- and MatriGrid^®^-cultured HepaRG cells in a concentration-dependent manner (Figure 3), demonstrating that measurement of secreted albumin can be used as a sensitive hepatofunctionality marker in drug testing, as other studies have observed [33]. MatriGrid^®^-differentiated HepaRG cells showed a higher sensitivity to APAP. Compared with monolayer cultures, a 60% reduction in albumin production was already detectable at 5 mM.

The newly developed 3D-ACAD device was now tested for its ability to detect albumin from the culture supernatants during 96 h perfusion of the HepaRG cultures with acetaminophen-containing culture medium or with growth medium alone (control). To monitor initial albumin levels, the HepaRG cell culture was continuously perfused with medium and a flow rate of 12 µL/min for 24 h. After the first automatic medium change and sampling run was completed, the cell culture was supplemented with 5 mM APAP or medium alone for another 72 h. Automatic medium change and sampling was carried out every 24 h.

**Figure 4:**
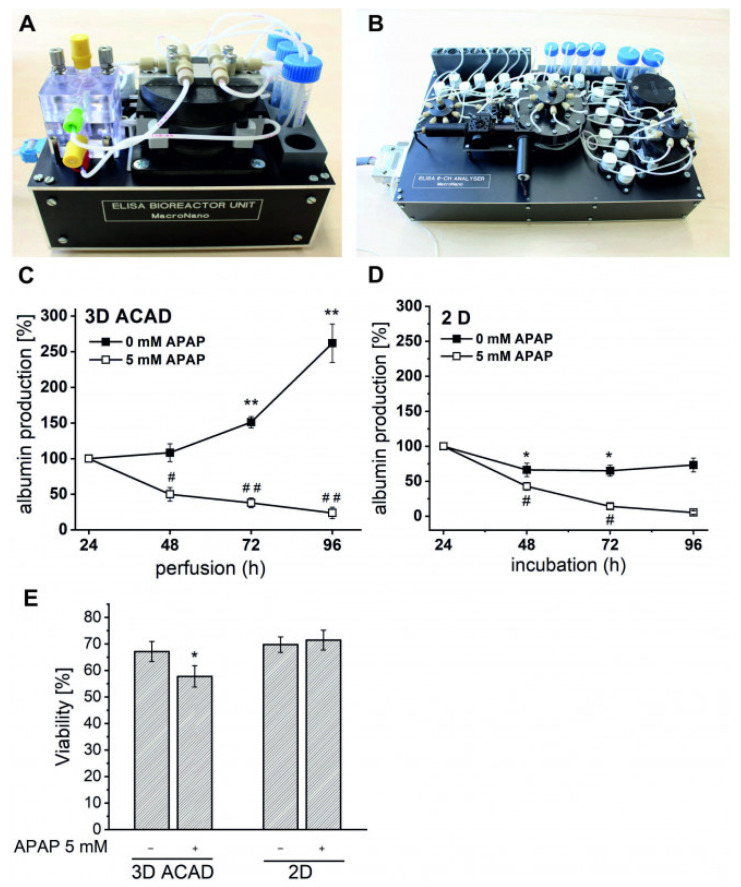
(**A**) The culture unit with the integrated bioreactor and MatriGrid^®^ scaffold for cell culturing and automated medium exchange. (**B**) The analysis unit for automated albumin concentration measurement. (**C**) Albumin production of HepaRG cells cultured in and measured with 3D-ACAD for 96 h. To monitor initial albumin levels, MatriGrid^®^ scaffolds with cells were perfused for 24 h with medium. Afterwards, perfusion occurred either with medium alone or medium supplemented with APAP. *0 mM APAP*: medium without APAP, *5 mM APAP:* medium with 5 mM APAP. Automated medium change and sampling for ELISA measurement was performed every 24 h by the 3D-ACAD. Albumin concentration was normalized to the total cell number (per million). Subsequently, albumin concentration at 24 h was set to 100% and albumin concentrations at 72 h were normalized to the value at 24 h. The mean values ± SE are shown; n = 4 experiments. Changes in albumin production in 3D-ACAD-cultured HepaRG cells perfused with medium or 5 mM APAP were statistically significant: ** *p* < 0.01; # *p* < 0.01. (**D**) Albumin production of HepaRG cells cultured in wells for 96 h. The same above-described procedure for 3D-ACAD was performed manually but with static well culturing and daily medium change. Albumin levels in medium supernatants were quantified with a commercial ELISA quantification kit and normalized as described above. The mean values ± SE are shown; n = 2 experiments. (**E**) Viability of cells after 96 h in the 3D-ACAD or wells were determined by measuring cell impedance using a CASY cell counter and analyzer system. Three-dimensional ACAD: n = 4; 2D: n = 2. The reduced viability of HepaRG cells in response to APAP was significant: * *p* < 0.05.

Albumin secretion increased significantly in control HepaRG cells over a total of 96 h of perfusion. There was a 51% increase at 72 h (** *p* < 0.01) and 161% increase at 96 h (** *p* < 0.01) compared with baseline albumin levels at 24 h (Figure 4C). In contrast, albumin secretion in APAP-treated HepaRG cells decreased to 49% (# *p* < 0.05) at 48 h, 38% (## *p* < 0.01) at 72 h and 24% (## *p* < 0.01) at 96 h.

The same experiment was performed with HepaRG cells cultured as monolayers (2D). HepaRG cells were first cultured with medium for 24 h to later detect basal albumin secretion. Subsequently, medium or medium containing 5 mM acetaminophen was added every 24 h for a period of another 72 h. The medium supernatants were analyzed for albumin content using a commercial ELISA quantification kit (Figure 4D). After 96 h incubation, we observed an 80% decrease in albumin production by monolayer HepaRG cells compared to the basal albumin secretion at 24 h. Repeated administration of acetaminophen to HepaRG monolayer cultures caused a decrease in albumin concentration to 61% at 48 h, 16% at 72 h and 0% albumin at 96 h.

Analysis of cell viability (Figure 4E) at the end of the experiment revealed that the 2D control HepaRG cells were viable in the same way as the 3D-ACAD-cultured control HepaRG cells despite sharply falling albumin levels with increasing culturing time. This implies that HepaRG cells cultured in 2D are likely to lose their ability to produce albumin under our experimental conditions and thus long-term drug-treatment experiments with this cell type under 2D conditions are not possible. In contrast, 3D-ACAD-cultured control HepaRG cells were viable and also after 96 h culturing in a differentiated state, which can be assumed due to the steadily increasing albumin secretion. This is supported by the fact that only in the 3D-ACAD system was a significant decrease in viability (10%; * *p* < 0.05) observed after 96 h of the acetaminophen treatment.

Comparing the HepaRG monolayer and 3D-ACAD culture methods, it becomes obvious that the automated perfusion of HepaRG cells with growth medium leads to a strong induction of albumin production and thus improves hepatofunctionality dramatically. In contrast, static monolayer culture of cells in growth medium for a time period of 96 h results in falling albumin levels compared to initial values and reduced hepatofunctionality. Repeated application of APAP initially appears to inhibit the secretion of albumin in both cell culture forms, but the true effect of acetaminophen on albumin production and cell viability is only observed in HepaRG cells in the 3D-ACAD system.

The data collected demonstrate that HepaRG cell functionality (measured by albumin production) over longer culturing periods is maintained only in the 3D-ACAD system. Accordingly, supply of nutrients to the cells and continuous removal of waste products of the cell culture by an automated perfusion system seem to be necessary to preserve the hepatic function of HepaRG cells in our experiments. Our 3D-ACAD system clearly shows an advantage over traditional cell cultivation systems; additionally, the automated fluidic and sampling gives the freedom to monitor the cellular status inside the incubator indirectly. Based on our data, the measurement of secreted albumin in an automated perfusion and analysis device (3D-ACAD) can be used in preclinical drug testing, as the decrease in albumin secretion in HepaRG cells by APAP treatment correlated with a decrease in cell viability. The data underline the importance of automated perfusion systems for maintaining hepatocyte functionality and CYP-based drug metabolism to better reflect the in vivo situation.

**Figure 5:**
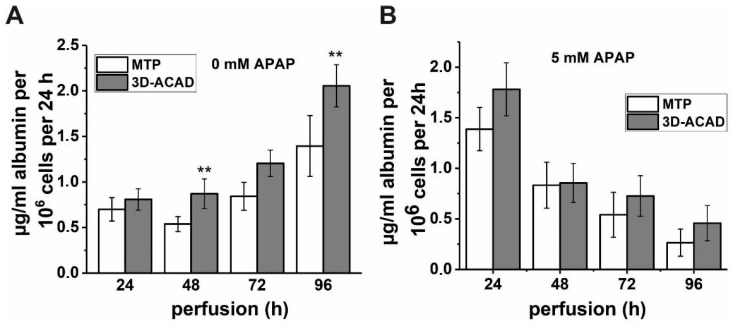
Comparison of albumin concentrations in medium supernatants without (**A**) and with (**B**) 5 mM APAP perfusion quantified with 3D-ACAD or with an ELISA quantification kit (MTP). The mean values ± SE are shown; n = 3 experiments; ** *p* < 0.01.

Finally, the accuracy of the adapted flow-through ELISA by 3D-ACAD was confirmed by measurement of the albumin concentrations of the 3D-ACAD samples using a traditional MTP ELISA (Figure 5). Therefore, medium supernatants collected from the 3D-ACAD device were subsequently analyzed with an ELISA quantification kit (MTP). We found significantly higher (*p* < 0.01) albumin levels measured with 3D-ACAD in the HepaRG controls at two measurement points (48 h and 96 h) and consistently slightly higher albumin levels with the 3D-ACAD measurement.

The reason for this is that two different albumin measurement methods with different system setups (flow-through and discontinuous) were used, as well as substrates with different readout specificities (ADHP fluorescence versus TMB absorbance). Thus, slight variation in measured albumin concentrations is to be expected. However, both types of ELISA (3D-ACAD versus MTP) detected comparable increases and reductions in albumin levels in the medium supernatants from cultured HepaRG cells. Thus, as part of the measurement accuracy, the values generated by 3D-ACAD could be verified by the MTP ELISA.

## 4. Discussion

We have introduced a novel 3D-ACAD culture and analysis system for alternative hepatotoxicity testing that integrates previously developed bioreactors with MatriGrid^®^ scaffolds [34]. We demonstrated the suitability of the scaffolds for 3D-culturing of HepaRG cells, with the resulting cultures being hepatofunctional and polarized: albumin and *bile canaliculi* were present and the expression of CYP2E1 and CYP3A4 was induced compared to undifferentiated 2D-cultured HepaRG cells.

We also showed that APAP reduces albumin secretion in HepaRG cells after a 24 h incubation, depending on the dosage, which underlines the possibility of using the albumin biomarker in hepatocyte toxicity studies.

Our 3D culturing unit is able to actively perfuse a 3D cell culture with different flow rates in long-term experiments, performs medium changes with repeated drug application and samples the culture medium for the analytic module. The culturing fluidic system stays closed during the experiment, which eliminates the risk of culture contamination. This increases the reliability of long-term experiments. The analytic module of the 3D-ACAD device is based on a flow-through adaptation of proven ELISA protocols, thus it provides stable and repeatable assay results. Verifying the albumin concentrations in the culture supernatants by the MTP ELISA demonstrated that the 3D-ACAD system provides very similar results, while saving manual labor related to medium change, sampling and performing time-consuming ELISA assays.

By comparing the results of the 3D-ACAD assay with the standard MTP ELISA, we have shown that HepaRG cultures in the 3D-ACAD system are hepatofunctional and, in contrast to 2D-cultured HepaRG cells, sensitive to a moderate repeated dose of acetaminophen (5 mM) after prolonged treatment (96 h). A parallel decrease in albumin secretion as well as cell viability after 96 h was observed. Two-dimensional-cultured HepaRG cells grown under our experimental conditions failed to maintain their hepatofunctionality, measured as secreted albumin, over a 96 h period. Therefore, the APAP-induced decrease in albumin content was more likely due to the reduced hepatocyte function of 2D-cultured HepaRG cells, whereas automated 3D perfusion of HepaRG cultures appears to improve hepatofunctionality compared to 2D-cultured HepaRG cells for use in repeated dose toxicity studies.

Our 3D-ACAD competes with a variety of published 3D-culture systems that are able to actively or passively perfuse and culture 3D organoids [35,36,37,38,39,40] and with commercially available culturing and perfusion systems (e.g., CellASIC, BellBrook Labs, Kiyatec, Cellek biotek). However, the novelty of our device is its ability to noninvasively measure toxicity by detection of biomarker molecules using on-demand, labor-saving flow-through ELISA. Devices of similar construction have so far only been shown by [16,17,18]. In contrast to these culturing systems, the 3D-ACAD design strictly avoids the usage of materials based on PDMS or hydrogels. PDMS material-based cell culturing devices experience biomarker- and drug-absorption and evaporation problems in the fluidic system [19,41]. In addition, hydrogel, which was used as support matrix for 3D cell cultures in these studies, in general slows down (or even traps) the diffusion of biomarkers and other molecules from cells to the medium [42]. Therefore, the implemented material becomes a limiting factor during long-term experiments, as do the biomarker concentrations in such complex culturing systems.

Similar to the standard MTP ELISA protocol, the 3D-ACAD system measures the sample and the standard curve in parallel (see Supplementary Materials). Thus, the readout data are always supplemented with a calibration standard. This is an advantage over single-channel sensors [17,18], which must be calibrated before the measurement. Since the properties of such sensors usually change with each subsequent assay, the measurements become gradually less accurate unless the sensor is periodically recalibrated. However, the calibration of single-channel sensors is time consuming—the whole assay must be performed several times in the case of a multipoint calibration. In contrast, the 3D-ACAD system provides the calibration standard curve simultaneously with the sample measurement; thus, no additional time is needed for calibration. The current limitation of the analytic module is its low throughput—only 7 PVC capillaries are available for sampling or standard measurement in a single run. The cleaning sequence has a relatively long duration of about three hours and thus requires further optimization.

The 3D-ADAC system developed in this work has great potential to be used in drug toxicity studies, the investigation of adverse drug reactions and drug–drug interactions and the simulation of disease models and hepatic immune reactions [43,44]. However, for real resemblance to the original liver tissue, co-cultivation with other essential cellular entities, such as sinusoidal liver endothelial cells, biliary epithelial cells, Kupffer cells and stellate cells, is required, which procedure is currently being developed by our research group. On the fluidic and automation side, the inclusion of multiple bioanalytical modules (e.g., a mass spectrometer for analysis of drug metabolites and an oxygen sensor) and parallel investigation of further liver co-culture-derived proteins (inflammatory cytokines, chemokines) by flow ELISA can be envisioned.

## 5. Conclusions

The 3D-ACAD system developed and described in this work represents a robust 3D cell culturing tool with automated medium change and automated biomarker analysis intended for daily laboratory use to support long-term experiments with minimal contamination risk and additional labor-saving benefits. Careful selection of the fluidic construction materials and optimization of fluidic components and operations allowed the construction of a flow-through-based ELISA system which can offer automated reliability compared to a traditional manual assay format. We demonstrated the functionality of this system with polycarbonate scaffold-cultured HepaRG organoids, which, due to their hepatofunctional properties, can be adequate for use in 2D-cultivated HepaRG cells in toxicity assays. In addition, automated 3D perfusion of HepaRG cultures appears to improve hepatofunctionality compared to 2D-cultured HepaRG cells for use in repeated-dose toxicity studies with longer experimental durations. Taking all these benefits into account, the 3D-ACAD system has the potential to be utilized at larger-scale levels with significantly higher throughput. This would greatly accelerate the development and testing of new drug therapies while simultaneously reducing cost and helping to improve existing alternatives to in vivo animal models for DILI evaluation.

## Data Availability

All supporting data is included in the paper.

## Supplementary Materials

Additional information regarding the microbioreactor, culture unit, analysis unit, ELISA automated assay procedure, cleaning procedure, fluorimeter and control unit can be found in the section on Material and Methods. The following are available online at https://www.mdpi.com/article/10.3390/bioengineering9050196/s1, Figure S1: Fluidic scheme of the culture unit, Figure S2: The analyzer module fluidics, Table S1: Valve settings for all operational modes of the culture system, Table S2: Timing of the complete assay sequence. References [22,34,45] are cited in the Supplementary Materials.
