# Supplementary material for: Automated Analysis of Acetaminophen Toxicity on 3D HepaRG Cell Culture in Microbioreactor
Source: Bioengineering (Basel). 2022 May 2;9(5):196. doi: 10.3390/bioengineering9050196 (PMC9137798; doi:10.3390/bioengineering9050196)
Supplement: Supplementary file 1 [file bioengineering-09-00196-s001.zip › bioengineering-1682260-supplementary.pdf]

Automated analysis of acetaminophen toxicity on 3D HepaRG cell culture in microbioreactor - Supplementary information

1. Micro bioreactor

The design and production of the micro bioreactor have been described recently [1]. Briefly, the externally perfused bioreactor used for 3D organotypic cell culture has a total fluid volume of 750 µl and outer dimensions of 35 x 30 x 46.8 mm (W x D x H). The housing consists of heat-resistant biocompatible PC components and is therefore autoclavable. Within the bioreactor, two fluid chambers are located above and below the inserted 3D cell carrier MatriGrid™ which are connected to an in- and out-flow channel to facilitate medium exchange and sample extraction. The inlet and outlet of the micro bioreactor are connected to medium containing tubes via 0.64 cm flangeless tube connectors (Upchurch Scientific, IDEX Health & Science LLC, USA). De-aeration of the fluid cycle is via an infusion port (B. Braun Melsungen AG, Melsungen, Germany).

2. Culture unit

The culture system comprises the micro bioreactor containing the MatriGrid™ scaffold [2] with the cell culture and the supporting platform. The culture unit contains one peristaltic pump (Boxer GmbH, OEM type 6131 with Pharmed tubing of inner diameter 0.51 mm) and four solenoid valves (Bio-Chem Valve Inc., 3 x type 075P3MP12-23B, 1x type 075P2NC12-23B). Additionally, the unit accommodates up to four 15 ml polypropylene tubes for the storage of cell culture medium or samples. For the perfusion loop the Pharmed tubing (Ismatec, type SC0339, inner diameter 0.51 mm) is used because it is required to withstand the autoclaving cycle. The rest of the unit uses C-Flex tubing with an inner diameter of 0.5 mm. The fittings are standard 0.64 cm-28 UNF PEEK types (Upchurch Scientific, IDEX Health & Science LLC, USA). The whole module is placed in the incubator with a controlled atmosphere (5% CO2) and temperature (37°C). The fluidic schematic of the culture unit is shown on the Figure S1. The corresponding valve setting for each mode of operation is listed in Table S1.

Table S1. Valve settings for all operational modes of the culture system.

	V25	V26	V27	V28
**Normal Perfusion**	OFF	OFF	OFF	OFF
**Medium Sampling- Phase I**	ON	OFF	OFF	ON
**Medium Sampling - Phase II**	OFF	ON	ON	OFF
**Medium Change**	ON	OFF	OFF	ON

3. Analysis unit

The analyzer unit consists of two peristaltic pumps (Boxer GmbH, OEM type 6131 with Pharmed tubing of inner diameter 0.51 mm), twenty one solenoid valves (Bio-Chem Valve Inc., 1x type 075P3MP12-23B and 20x type 075P2NC12-23B), one 9-port manifold (Upchurch, type P-191-01) with standard 0.64 cm-28UNF PEEK fittings, polypropylene tubes for all reagents and standards/samples and three custom made FEP manifolds (2x 9-port and 1x 6-port, welded FEP tubing: VICI JR-T-6802, 0.16 cm x 0.5 mm). During analysis, the analyte and other reactants are pumped through the fluidic pathways (FEP / C-Flex tubing) controlled by valves and peristaltic pumps into the respective PVC capillary (20 µl, 1.8 mm OD, 0.95 mm ID, SC-Sanguia Counting 100024) where the detection takes place. Further hardware components include a capillary revolver powered by NEMA17 stepper motor and the fluorescence detector (standard optical components provided by Thorlabs and Edmund Optics. The purpose of the sample revolver is to position the

selected capillary to the focus point of the optical setup. A potential displacement error is eliminated by continuously measuring the fluorescence while the capillary is passing through the focus point. The measured curve is then evaluated instead of a single point. As the integral part of the analyzer unit, the fluorescence detector uses the 10 mW green 532 nm DPSS laser (Thorlabs, DJ532-10) as the excitation light source and the photodiode (Osram, BPX61) for the emission light detection. To limit the detection to the resorufin emission (peak at 585 nm) spectrum, the 592 nm band-pass filter with OD 6 was used (Edmund Optics, NT67-020). The fluidic schematic of the analysis unit is shown on the Figure S2.

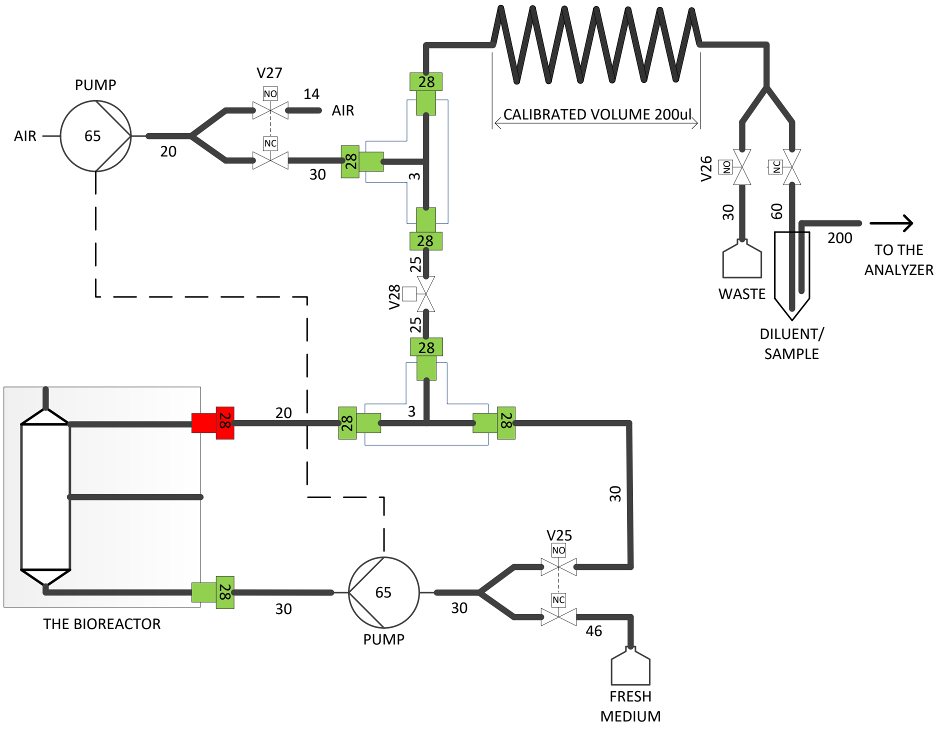

Figure S1. Fluidic scheme of the culture unit (the numbers show the dead volume in µl of the fluidic components).

4. ELISA automated assay-procedure

Before starting the automated assay, new PVC capillaries are inserted into the analysis revolver and six sample reservoirs were filled with the prepared albumin concentration standards. The sample reservoir was connected to the sample port of the culture module. Washing solution (25 ml) was added to the washing reservoir, and reservoirs containing capture antibody solution or blocking solution were filled with 1 ml of the respective reagent. The QuantaRed™ Enhanced Chemifluorescent HRP Substrate (Thermo Scientific #15159) was used as the substrate for the fluorescence quantitation. As the HRP conjugate and HRP substrate solution are unstable, the corresponding reservoirs are filled during the assay with freshly prepared solution approximately 15 min before the pumping operation. During analysis, the analyte and other reactants are pumped through the fluidic pathways (FEP / C-Flex tubing) controlled by valves and peristaltic pumps into a PVC capillary where the detection takes place. Table S2 lists the complete analysis procedure divided to single steps. The timing information for each sequence step is also provided.

Table S2. Timing of the complete assay sequence.

Sequence step	Description	Step time [min]	Total time [min]

1	Pumping capture antibody to capillaries	4.46	4.46
2	Coating incubation	15	19.46
3	Washing	9.7	29.16
4	Pumping blocking buffer to capillaries	4.46	33.62
5	Blocking incubation	30	63.62
6	Washing	9.7	73.32
7.1	Pumping Sample 1 to capillary 1	1.38	74.7
7.2	Cleanup after pumping sample 1	3	77.7
7.3	Pumping Sample 2 to capillary 2	1.38	79.08
7.4	Cleanup after pumping sample 2	3	82.08
7.5	Pumping Sample 3 to capillary 3	1.38	83.46
7.6	Cleanup after pumping sample 3	3	86.46
7.7	Pumping Sample 4 to capillary 4	1.38	87.84
7.8	Cleanup after pumping sample 4	3	90.84
7.9	Pumping Sample 5 to capillary 5	1.38	92.22
7.10	Cleanup after pumping sample 5	3	95.22
7.11	Pumping Sample 6 to capillary 6	1.38	96.6
7.12	Cleanup after pumping sample 6	3	99.6
7.13	Pumping Sample 7 to capillary 7	1.38	100.98
7.14	Cleanup after pumping sample 7	3	103.98
8	Sample incubation	15	118.98
9	Washing	9.7	128.68
10	Pumping detection antibody to capillaries	4.46	133.14
10.1	Cleanup after det. Antibody pumping	4.63	137.77
11	Detection antibody incubation	15	152.77
12	Washing	14.55	167.32
13	Pumping substrate to capillaries	4.46	171.78
14	Incubation and fluorescence measurement	21.35	193.13

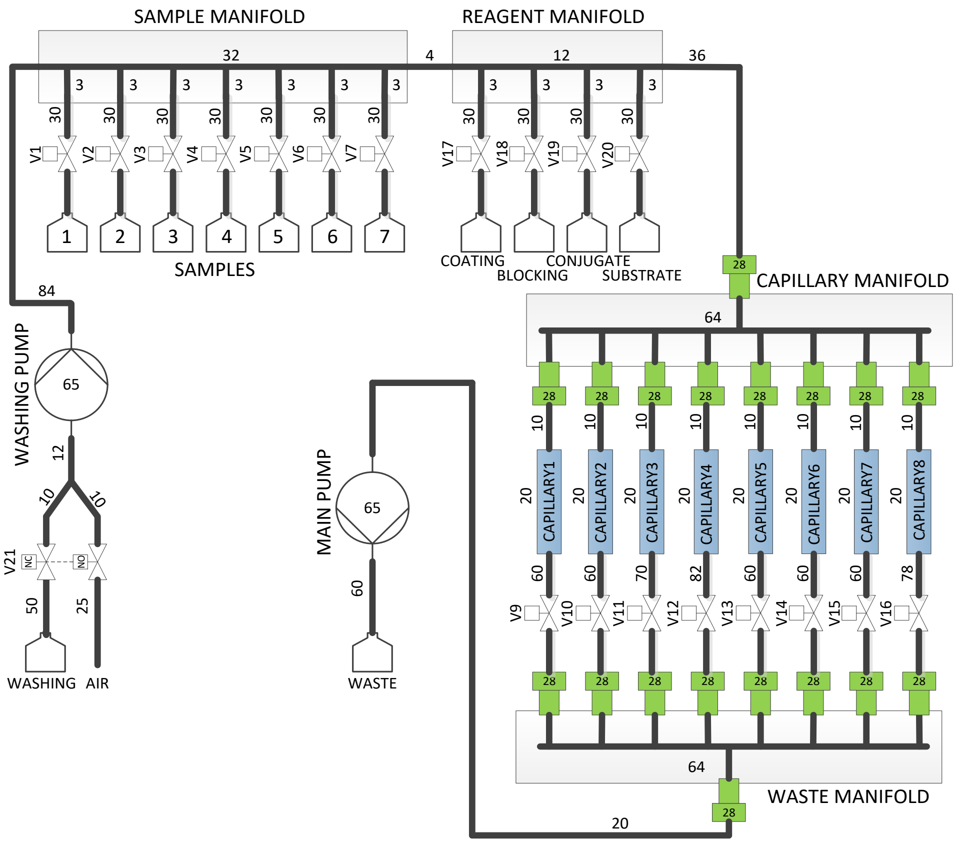

Figure S2. The analyzer module fluidics (the numbers show the dead volume in μl of the fluidic components.

5. Cleaning procedure:

The cleaning procedure between assays begins by washing of all fluidic pathways with 1M NaOH as the first step, followed by washing with protein desorption solution (2% citric acid and 0.5% SDS in 70% ethanol). The third cleaning step is washing the fluidics with 70% ethanol, followed by the final washing with DI water. This four step cleaning procedure proved to be very efficient and fluorescent background levels of 0.5% were achieved after single cleaning procedure. The cleaning procedure takes about 3 hours.

6. Fluorimeter

The fluorimeter was designed to measure the fluorescence from a cylindrical shaped space with the length of 8mm and diameter of 1mm. The excitation and emission filters were optimized for the fluorescence measurement of resorufin. The emission sensing axis is at the right angle with the excitation beam axis. The fluorimeter uses construction components of the ½ inch lens tube system marketed by Thorlabs.

6.1. Excitation optics description:

The 10mW green DPSS laser was used as the source of excitation light (DJ532-10, Thorlabs). The laser beam is first expanded using the sapphire ball lens (diameter 0.5mm, type 46117, Edmund Optics) and collimated into parallel beam using the plano-convex lens (LA1540, Thorlabs). The beam is then passed through the 50:50 beam splitter (BS010, Thorlabs) which diverts the portion of the beam to the feedback photodiode (BPW34, Vishay) of the laser driver circuit to keep the optical power fluctuation below 0.01% required for the operation within the dynamic range of four orders of magnitude. The other portion of the excitation beam is focused by the cylindrical lens (type 46194, Edmund Optics) to the center of the modified cage cube (SC6W, Thorlabs) where the capillary is

aligned in the focal line of the excitation beam by the rotary sample holder. The length of the capillary interior which is illuminated by the excitation beam is about 8mm.

6.2. Emission optics description:

A portion of the resorufin emitted light is collimated into a parallel beam using the cylindrical lens (type 46194, Edmund Optics), which is passed through the suitable optical filter (type 67020, Edmund Optics). The filtered light is focused by the plano-convex lens (LA1540, Thorlabs) to the radiant sensitive area of the photodiode (BPX61).

The diver circuit is based on a dedicated integrated circuit (iC-WKN, iC-Haus). The photodiode BPX61 works in photovoltaic mode and it is connected to the transimpedance amplifier with the gain of 107. The photodiode is not biased which eliminates the dark current and the internal diode capacitance stays at constant (zero) potential, therefore its influence on the detector speed is minimal. The amplified signal is digitized inside the control unit using the 16-bit ADC converter (AD7794).

7. Control unit

The core of the control unit makes use of the 32-bit microcontroller (STM32F103ZET, STMicroelectronics) which is based on the ARM CORTEX-M3 architecture. Interfacing to optional host system is possible using the USB or serial interfaces. The corresponding printed circuit board (PCB) was designed using four electrical layers and has the size 160 x 200mm. The whole system is powered by single 12V source. The code for the control unit was written in ANSI C programming language and it was compiled for the ARM CORTEX-M3 architecture. The latest source code version v1.09 contains approximately 10000 lines of code excluding used libraries. Additionally, the open source bootloader OpenBLT [3] was used to simplify the firmware update procedure.

References

1. Fernekorn, U.; Hampl, J.; Weise, F.; Augspurger, C.; Hildmann, C.; Klett, M.; Läffert, A.; Gebinoga, M.; Weibezahn, K.-F.; Schlingloff, G., et al. Microbioreactor design for 3-D cell cultivation to create a pharmacological screening system. *Engineering in Life Sciences* **2011**, *11*, 133-139, doi:10.1002/elsc.201000145.
2. Borowiec, J.; Hampl, J.; Gebinoga, M.; Elsarnagawy, T.; Elnakady, Y.A.; Fouad, H.; Almajhadi, F.; Fernekorn, U.; Weise, F.; Singh, S., et al. Thermoforming techniques for manufacturing porous scaffolds for application in 3D cell cultivation. *Materials Science and Engineering: C* **2015**, *49*, 509-516, doi:http://dx.doi.org/10.1016/j.msec.2015.01.002.
3. Feaser *OpenBLT GNU GPL Bootloader*, https://www.feaser.com/en/openblt.php: 2017.
